# Cardiotoxicities of 5-Fluorouracil and Other Fluoropyrimidines

**DOI:** 10.1007/s11864-020-0719-1

**Published:** 2020-03-19

**Authors:** Taro Shiga, Makoto Hiraide

**Affiliations:** 1 Department of Onco-Cardiology/Cardiovascular Medicine, The Cancer Institute Hospital Of Japanese Foundation for Cancer Research, 3-8-31 Ariake, Koto-ku, Tokyo, 135-8550 Japan; 2 Department of Pharmacy, The Cancer Institute Hospital Of Japanese Foundation for Cancer Research, 3-8-31 Ariake, Koto-ku, Tokyo, 135-8550 Japan

**Keywords:** Fluoropyrimidine, 5-Fluorouracil, Endothelial dysfunction, Coronary spasm, Myocardial cell damage

## Abstract

Fluoropyrimidine (FP) is used to treat a wide range of cancers; however, it is associated with drug-induced vascular toxicity, as well as angina pectoris and coronary spasm. FP has been administered for many years, although the incidence, mechanisms, and appropriate methods for managing its associated cardiovascular toxicities have not been clarified, and the management of these complications has not been standardized. This lack of evidence is not limited to FP. Many trials of anticancer agents have been conducted, excluding patients with heart diseases. Hence, there is a paucity of epidemiological data on cardiovascular adverse events caused by anticancer agents. There have been remarkable improvements in cancer treatment in recent years, with consequent improvements in prognosis. In this context, new cardiovascular toxicities related to new drugs have emerged. We are now compelled to respond to cardiovascular adverse events despite the lack of evidence regarding optimal management. The result has been establishment and rapid maturation of the new academic field of cardio-oncology. Despite the relative lack of evidence, we must review small pieces of evidence that have accumulated to date and make the utmost efforts to provide patients with effective evidence-based medical care. Simultaneously, we urgently need randomized clinical trials to build strong evidence.

## Introduction

According to the WHO, cancer incidence is predicted to increase by about 70% over the next 20 years [1]. While survival rates of those diagnosed with cancer are expected to significantly improve, complications related to cancer treatment are also anticipated. Remarkable advances in cancer treatment have led to a significant decline in cancer-related mortality; concomitantly, non-cancer diseases have become prominent modifiers of quality of life as well as overall survival rates.

In particular, cardiovascular diseases such as angina pectoris and heart failure significantly alter overall morbidity and mortality. The need for appropriate interventions for cardiovascular diseases in cancer patients and cancer survivors has given rise to the medical specialty of cardio-oncology [2].

Anthracycline, an important anticancer agent with a long track record, and trastuzumab, used for breast and gastric cancers, cause cardiotoxicity [3]. Many other molecular-targeted drugs that have become available in recent years, especially vascular endothelial growth factor pathway inhibitors, also cause cardiovascular toxicity. Fluoropyrimidine (FP), including 5-fluorouracil (5-FU) and capecitabine, are also notable in this regard. The cardiovascular toxicity associated with FP remains a critical issue. There is no solid evidence worldwide that allows risk stratification, even though the agent has been used for many years.

FP is an antimetabolite agent used to treat solid tumors such as colon, breast, and head and neck cancers. It possesses a structure similar to that of substrates and enzymes required for DNA replication. Subsequent to cellular uptake, FP becomes active by each and exerts its antitumor effects by inhibiting DNA and RNA synthesis. 5-FU possesses a structure in which a hydrogen at position 5 of the pyrimidine ring is substituted by fluorine. This drug was generated because uracil is readily taken up into tumor cell DNA. 5-FU is metabolized to 5-fluorodeoxyuridylate, suppressing DNA synthesis by inhibiting thymidylate synthase. 5-FU is also converted to 5-fluorouridine triphosphate, which is incorporated into RNA and inhibits RNA synthesis [4]. Capecitabine is an oral prodrug of 5-FU designed to be converted selectively in tumors. It is rapidly absorbed from the gut as an unchanged drug and is converted to 5′-deoxy-5-fluorocytidine via hydrolysis by carboxylesterase in the liver. It is further converted to an active form of 5-FU by thymidine phosphorylase present at high levels in tumor tissues and exerts antitumor effects specifically in tumors. Other FP drugs include S1 (tegafur/gimeracil/oteracil) and UFT (tegafur/uracil). Although drug indication criteria varies among countries, these drugs are sometimes used as alternatives to 5-FU if they are indicated for a particular type of cancer [5].

Cardiovascular adverse events associated with FP, especially with FU, include angina with coronary artery spasm and ischemic heart disease secondary to coronary artery thrombosis. In addition to ischemic heart diseases, there have been reports of direct myocardial damage, including more serious cardiovascular toxicity [6–9]. 5-FU and capecitabine are generally well tolerated, and the most common adverse events are bone marrow suppression and gastrointestinal and skin toxicities. Although both drugs rarely induce cardiovascular toxicity, various conditions, including acute coronary syndrome, arrhythmia, cardiomyopathy, heart failure, hypertension and hypotension, shock, and sudden cardiac death, can potentially occur [6].

In this review, we present an updated overview of FP-induced cardiovascular toxicity, its mechanisms, pathophysiology, and available treatment options.

## Epidemiology and risk factors

Incidence of FP-related cardiovascular toxicity ranges from 1 to 19% [10], and mortality has been reported to be 2.2–13.3% [11–18]. This wide range of incidence may reflect the differences among risk profiles of patient groups in each study and differences in drug administration schedules (Table 1) [11, 13, 21, 27, 28, 40, 41]. FP-related cardiovascular toxicity is enhanced by simultaneous chest radiotherapy [42], multidrug chemotherapy [41], history of coronary artery disease, structural heart disease such as valvular disease, and various cardiomyopathies [11, 40, 43]. Other known risk factors for cardiovascular diseases, including smoking, diabetes, hypertension, dyslipidemia, and obesity, may exacerbate FP-related cardiovascular toxicity. Nevertheless, it is currently impossible to predict FP-related cardiovascular toxicity with certainty based on the presence of these risk factors [13]. Appropriate interventions should be made to manage these traditional risk factors of cardiovascular disease.

**Table 1:** Studies of FP-related cardiovascular toxicities

Incidence of cardiotoxicity according to chemotherapy regimens						
Author	Primary cancer	Chemotherapy regimen	Number of patients	Overall 5-FU-induced cardiotoxicity incidence (N)	Signs and symptoms	Timing of onset
Jin et al. [19]	Gastric cancer	5-FU-containing regimens	129	29.5% (38)	Chest pain	Not reported
					Heart failure	
					Atrial fibrillation	
					Acute myocardial infarction	
					Sinus arrhythmia	
					Palpitation	
					Ventricular premature beat	
					Atrioventricular block	
					QT interval prolongation	
					Abnormal ST and T waves	
Kwakman et al. [20]	Colorectal cancer	Capecitabine	1973	5.9% (117)	Cardiac ischemia	Note reported
		CAPIRI			Arrhythmia	
		CAPOX			Chest pain	
		CAPOX bevacizumab			Heart failure	
					Cardiac death	
Turan et al. [21]	Colorectal cancer	5-FU-containing regimens	32	12.5% (4)	Angina	During the first hours of continuous 5-FU infusion
	Gastric cancer				ECG changes	Just after minute after the bolus 5-FU administration
	Pancreatic cancer					
	Head and neck cancer					
Płońska-Gościniak et al. [22]	Colorectal cancer	LF1	25	unknown	Prolonged QT interval	Not reported
		XELOX			Worsening in left ventricular	
		Capecitabine			Functional parameters by tissue	
		FOLFIRI			Doppler	
Polk et al. [23]	Breast cancer	Capecitabine	452	4.9% (22)	Chest pain	In the first cycle: 50% (11)
					Dyspnea	In the second cycle: 18% (4)
					Palpitations	In the third cycle: 14% (3)
					Atrial fibrillation	In the fourth cycle: 4.5% (1)
					ST deviations	
					Negative or fluctuating T-waves	
					Cardiac arrest	
Lestuzzi et al. [10]	Gastric cancer	5-FU-continuous infusion	228	10.3% (37)	Angina	Not reported
	Head and neck cancer	TCF			ECG changes	
	Colorectal cancer	CDDP 5-FU (± RT)				
		FOLFOX				
		FOLFIRI				
Jensen et al. [7]	Colorectal cancer	FOLFOX4	106	8.5% (9)	Angina	Not reported
Khan et al. [24]	Unknown	5-FU-containing regimens	301	19.93% (60)	Angina	Not reported
					Bradycardia	
					Ventricular tachycardia	
					Sudden death	
					Cardiac arrest	
Koca et al. [25]	Gastric cancer	Capecitabine mono	52	34.6% (18)	New-onset cardiovascular symptoms	1 h - 24 days
	Colorectal cancer	Capecitabine containing regimens		11.5% (6)	New-onset physical sigins	
	Breast cancer			32.6% (17)	New-onset ECG signs	
Salepci et al. [26]	Gastric cancer	5-FU-containing regimens	31	unknown	Decrease in mean brachial artery diameter	Immediately after first 5-FU treatment
	Colorectal cancer					
Kosmas et al. [13]	Colorectal cancer	Capecitabine	644	4.03% (26)	Angina	Not reported
	Breast cancer	LV5-FU2			Palpitations	
	Head and neck cancer	CDDP 5-FU			Sudden death	
		MMC 5-FU			ECG changes	
Yilmaz et al. [27]	Colorectal cancer	LV5-FU2	27	7.4% (2)	Angina	Not reported
	Gastric cancer				Decrease in mean heart rate	
	Esophageal cancer				Increase in the number and complexity of premature complexes	
Holubec et al. [28]	Colorectal cancer	De Gramont regimen	42	unknown	Laboratory signs of coronary	Not reported
		FOLFIRI			Ischemia	
					Laboratory signs of heart failure	
Tsibiribi et al. [29]	Colorectal cancer	5-FU	1350	1.2% (16)	Angina	Not reported
	Gastric cancer				Heart failure	
	Oesophageal cancer				ECG changes	
	Pancreatic cancer					
Jensen et al. [30]	Colorectal cancer	Capecitabine	668	4.3% (29)	Angina	Capecitabine (XELOX): median 4 days (min: 2 days - max: 15 days)
	Gastric cancer	XELOX			Heart failure	5-FU Mayo: median 5 days (min: 3 days - max: 7 days)
		TCX				De Gramont regimen, FOLFOX4: median 3 days (min: 2 days - max: 6 days)
		De Gramont regimen				
		5-FU Mayo				
		FOLFOX4				
Tsavaris et al. [31]	Colorectal cancer	5-FU-containing regimens	522	3.8% (20)	Acute myocardial infarction	Not reported
	Head and neck cancer				Ischemia	
Ceyhan et al. [32]	Colorectal cancer	HD-LV5-FU	37	5.4% (2)	Transthoracic echocardiography and cyclic variation of integrated backscatter (CVIBS)	CVIBS: significantly decreased at the 48th hour of treatment
	Gastric cancer					
	Head and neck cancer					
	Breast cancer					
Ng et al. [33]	Colorectal cancer	XELOX	153	6.5% (10)	Angina	Median cycle1 dayto (min: cycle1 day4-max: cycle4)
					Sudden death	
					Heart failure	
Meydan et al. [34]	Gastrointestinal	De Gramont regimen	231	3.9% (9)	Acute coronary syndrome	1–16 days
	Breast cancer				Congestive heart failure	
	Head and neck cancer				Atrial fibrillation	
Barutca et al. [35]	Gastrointestinal	5-FU-containing regimens	28	0.0% (0)		Not reported
Oztop et al. [36]	Gastrointestinal	LV5-FU2	22	Clinically evident cardiac event: 0.0% (0) QT interval prolongation: unknown	QT interval prolongation	QT interval prolongation: as early as 24 h
Sudhoff et al. [37]	Gastrointestinal	5-FU-containing regimens	30	unknown	Contraction brachial artery	Not reported
	Lung cancer					
	Lymphoma					
	Head and neck cancer					
Wacker et al. [12]	Gastrointestinal	5-FU-containing regimens	102	18.6% (19)	Angina	Within 24 h
	Head and neck cancer					
Van Cutsem et al. [38]	Colorectal cancer	5-FU (593)	1425	3.2% (46)	Chest pain	Not reported
	Breast cancer	Capecitabine (832)			Sudden death	
					Heart failure	
Balloni et al. [39]	Colorectal cancer	5-FU-containing regimens	25	8.0% (2)	Tachycardia	Not reported

CAPIRI: capecitabine + irinotecan; CAPOX, XELOX: capecitabine + oxaliplatin; CDDP 5-FU: cisplatin +5-FU; De Gramont regimen: leucovorin + 5-FU IV bolus and 5-FU continuous IV infusion; FOLFIRI: irinotecan + leucovorin +5-FU IV bolus and 5-FU continuous IV infusion; FOLFOX: oxaliplatin + leucovorin + 5-FU IV bolus and 5-FU continuous IV infusion, HD-LV5-FU: high-dose leucovorin + 5-FU. LF1: leucovorin + 5-FU; LV5-FU2: leucovorin + 5-FU IV bolus and 5-FU continuous IV infusion; MMC 5-FU: mitomycin + 5-FU; TCX: capecitabine + carboplatin + docetaxel; TPF: docetaxel + cisplatin + 5-FU; 5-FU Mayo: isovorin + 5-FU

In a review of 377 cases of FP-related cardiotoxicity, while only 14% of patients had a history of heart disease, 37% were found to have known risk factors for heart diseases [8], of which smoking was the most common. Nevertheless, there is no strong evidence that the traditional risk factors for cardiovascular diseases are significantly involved in the induction of FP-related cardiovascular toxicity [6, 13, 33, 41]. Despite the fact that advanced age is a risk factor for FP-related cardiovascular toxicity, there is no solid supportive evidence [8, 30, 40]. Taken together, the data suggest that there is no solid evidence available to enable risk stratification that selects patients for whom FP administration should be discontinued.

Concomitant use of FP with cisplatin and leucovorin increased the incidence of 5-FU-related cardiovascular toxicity [13, 24]. There have been no such data regarding anthracycline, a drug noted for cardiotoxicity, in terms of increased incidence of 5-FU-related cardiovascular toxicity.

5-FU is considered a radiosensitizer in patients receiving radiation therapy and concomitant chemoradiotherapy; there have been reports of possible involvement of 5-FU in the development of cardiovascular complications by accelerating small vessel thrombosis [8, 30].

The incidence of cardiovascular toxicity attributable to 5-FU may depend on the administration route. Cardiovascular toxicity occurs more commonly when administered by continuous intravenous infusion over many hours [13, 24, 30, 34, 44]. The incidence of cardiovascular toxicity ranged between 1.6 and 3.0% when administered by bolus intravenous injection [40, 45], whereas the range was 2.0–18% when administered by continuous intravenous infusion for 5 days or longer [8, 11, 13, 34, 41]. The reason for the lower incidence of toxicity with the bolus injection method compared to continuous infusion method may relate to the short half-life of 5-FU, which is 15–20 min [46]. During treatment with the FOLFOX regimen, in which oxaliplatin and leucovorin are added to 5-FU infusion, the incidence of chest pain was reported to be about 9% [47]. The incidence of cardiovascular toxicity attributable to capecitabine, an oral prodrug of 5-FU, has been reported to be 3–9%, almost the same as that attributable to continuous intravenous injection of 5-FU [13, 33, 46]. Capecitabine requires the same level of attention regarding cardiovascular toxicity complications as does 5-FU. Of a total of 377 patients that exhibited FP-related cardiovascular toxicity, 72% had administered the drug by continuous intravenous infusion, 23% received bolus intravenous injection, 3% received non-long-term continuous intravenous infusion, and 2% received oral administration [8].

Several factors have been identified as major issues regarding FP-related cardiovascular toxicity. It is difficult to summarize data from the large number of published reports. The definitions of FP-related cardiovascular toxicity of each report are often based on different criteria; many are case reports; many others lack accurate data in line with current methods, i.e., combination therapy with other cardiotoxic anticancer agents rather than FP monotherapy [48].

## Clinical manifestations

The most common symptom related to 5-FU-induced angina is chest pain. There are also reports of palpitation, shortness of breath, and pleural pain. Symptoms appear at rest or on exertion [10]. Patients may suffer asymptomatic myocardial ischemia [11, 14, 43]. Because 5-FU and capecitabine are primarily administered on an outpatient basis, asymptomatic electrocardiogram (ECG) abnormalities and cardiovascular toxicity may be overlooked.

There is no difference between 5-FU-induced cardiovascular toxicities and those caused by capecitabine [33]. Angina is the most common form of cardiovascular toxicity. Saif et al. reported 377 patients with FP-related cardiovascular toxicities and found that angina accounted for 45%; myocardial infarction, 22%; arrhythmia, 23%; acute pulmonary edema, 5%; heart failure, 2%; and cardiac arrest and pericarditis, 1.4% each [8]. Associated arrhythmias included bradycardia, atrial fibrillation, ventricular tachycardia, and ventricular fibrillation [9, 49–51]. There have also been reports of takotsubo cardiomyopathy following FP drug administration [52, 53].

FP-related cardiovascular toxicity usually develops during the first cycle of administration [8, 33, 41, 54], mostly within 72 h after administration. An analysis of 102 consecutive patients who were treated with 5-FU showed that reversible angina attacks lasting up to 12 h occurred within 24 h after the start of FU administration in 19 patients (19%); these lasted for 12 h at most after discontinuation of FP [12]. Symptoms such as chest pain improved relatively rapidly after discontinuation of FP; FP-related cardiovascular toxicity was generally reversible.

## Detection of cardiovascular toxicities

No diagnostic methods have been established by which FP-related cardiovascular toxicity can be specifically diagnosed. To correlate cardiovascular events with FP treatment, causal relationships should be clarified based on clinical course, e.g., increased incidence of cardiovascular events occurring during treatment with FP and reproducibility of cardiovascular events upon re-administration of FP.

### Biomarkers

Biomarkers are commonly used in cardiovascular clinical practice, including those that indicate myocardial injury, such as troponins T and I, creatine kinase myocardial band, and those that indicate cardiac load, such as B-type natriuretic peptide. These biomarkers are not highly sensitive to FP-related cardiovascular toxicity. Nevertheless, as cardiovascular toxicity develops, levels of these biomarkers increase abnormally [9, 52, 55]. In asymptomatic patients, Holter-ECG monitoring may be performed upon incidental findings of abnormal biomarker test results and can lead to successful diagnosis of FP-related cardiovascular toxicity.

Jensen et al. examined biomarkers related to the coagulation system following FP administration. They measured thrombus-related biomarkers in patients with colorectal cancer, focusing on vascular endothelial damage by FP and subsequent thrombus formation. The authors found significant increases in D-dimer and von Willebrand factor (wWF) levels, while levels of coagulation factors II, VII, and X significantly decreased [7]. Despite the fact that FP may have a coagulation-accelerating effect, there is insufficient evidence to suggest that they are useful biomarkers for FP-related cardiovascular toxicity. Another study reported that levels of coagulation biomarkers such as D-dimer did not change significantly after FP administration [35].

Other potential biomarkers of FP-related cardiovascular toxicity include heart-type fatty acid-binding protein [21] and angiotensin II [26], both of which are released from cardiomyocytes into the bloodstream upon myocardial damage. Nevertheless, none of these papers demonstrated meaningful objective data of these biomarkers.

### Electrocardiogram

ECG abnormalities are not highly sensitive for detecting abnormalities due to FP-related cardiovascular toxicity; nevertheless, supraventricular arrhythmias including arterial fibrillation [56], ventricular tachycardia, and ST-T wave abnormalities can be listed as ECG abnormalities attributable to FP-related cardiovascular toxicity. Of these, ST-T wave abnormalities reflecting myocardial ischemia due to angina pectoris are seen most frequently. There are asymptomatic cases with ST-T wave abnormalities [11, 14, 43], and there are likely to be more cases of asymptomatic myocardial ischemia that have not been examined in clinical settings. There are also reports of QT prolongation, and although rare, cases of torsade de pointes have also been reported [9, 12, 36].

Holter-ECG is useful for detecting 5-FU-related cardiovascular toxicity, especially ST-T wave abnormalities and arrhythmias [12, 27, 35]. In fact, we cared for a patient in whom we successfully detected an ST-T wave abnormality using Holter-ECG (Table 2).

**Table 2:** Two cases of 5-FU-related coronary spasm those we recently experienced at The Cancer Institute Hospital of Japanese Foundation for Cancer Research

Case	Age (years)	Sex	Primary cancer	Chemotherapy regimen	Cardiotoxicity	Signs and symptoms	Diagnosis	Treatment	Onset time	Time to onset	Outcome
1	62	Male	Rectal cancer	Modified FOLFOX6	Angina (Coronary Spasm)	Chest pain	Electrocardiography	Sublingual glyceryl trinitrate	Course 1 day 2	24–35 h	The chemotherapy regimen was changed → irinotecan monotherapy
						High blood pressure		Nicorandil			
2	77	Female	Rectal cancer	FOLFIRI plus bevacizumab	Angina (Coronary Spasm)	Chest pain	Electrocardiography	Sublingual glyceryl trinitrate	Course 1 day 2	36 h	The chemotherapy regimen was changed → S-1 + irinotecan + bevacizumab
						High blood pressure					

### Imaging techniques

#### Echocardiogram

Echocardiography is not highly sensitive for detecting abnormalities caused by FP-related cardiovascular toxicity; nevertheless, echocardiogram can detect diffuse or focal left ventricular hypokinesis or decreased ejection fraction (EF) caused by FP. Wacker et al. found decreased EF in 10.5% of patients with symptomatic acute FP-related cardiovascular toxicity [12]. Nevertheless, these findings of decreased cardiac function are atypical, and normal findings can often be seen. In several reports, no significant changes of diastolic or systolic dysfunction attributable to FP administration were confirmed according to conventional echocardiographic parameters [5, 32, 35, 39]. By contrast, Turan et al. reported that 18.7% of patients who were treated with 5-FU had significantly lower systolic and diastolic function after one cycle of 5-FU administration and that the Tei index was useful for detecting latent myocardial damage [21]. There was also a study that found selected tissue Doppler parameters significantly decreased after undergoing chemotherapy including 5-FU [22]. In general, the literature on this issue is complicated by variations in data accuracy among various echocardiography techniques and facilities, which results in insufficient evidence regarding the significance of these findings.

#### CCTA and catheterization

In the context of ST-T abnormalities and detection of cardiac enzymes such as troponin, it is necessary to exclude coronary artery lesions. The high negative predictive value of coronary computed tomography angiography (CCTA) in detecting coronary artery disease is well known. CCTA is relatively non-invasive and generally highly significant in the context of low risk patients. If a coronary artery lesion is detected using CCTA, detailed evaluation with high diagnostic significance such as catheterization is then proposed. Catheterization is indicated expediently for patients requiring further detailed examinations after CCTA for patients with heart failure, recurrent angina attacks, serious arrhythmias such as ventricular tachycardia and ventricular fibrillation, as well as for patients at high risk for coronary artery lesions such as cardiogenic shock. Coronary spasm is often involved in FP-related angina, and in many cases, coronary artery lesions are not detected or only mild stenotic lesions are detected [9, 51, 57, 58]. In patients where significant coronary artery stenosis is not detected, if other various findings suggest the presence of a myocardial ischemic event (e.g., reproducible chest pain by FP administration, abnormal rise of biomarker levels after FP administration, or detection of obvious ST-T wave abnormalities and confirmation of improvement of ST-T wave abnormalities after discontinuation of FP administration), a speculative diagnosis of FP-related coronary artery event is sometimes made. In the patient we treated, the diagnosis was made by a similar method (Table 2).

In some patients, coronary angiography only detects moderate stenotic lesions in coronary arteries, making it difficult to determine whether the chest pain is attributable to a coronary artery event. In such cases, to make a definitive correlation with coronary spasm, provocative testing using ergonovine or acetylcholine is sometimes considered. Provocative testing is invasively risky; therefore, determining its indication requires careful consideration. In patients with coronary spasm symptoms with high clinical certainty in whom significant stenosis of coronary arteries are not found, and no abnormalities of ST-T wave are detected, provocative testing should be performed after obtaining informed consent and thorough examination of the risk-benefit.

## Pathogenesis and mechanisms of cardiovascular toxicities

**Fig. 1:**
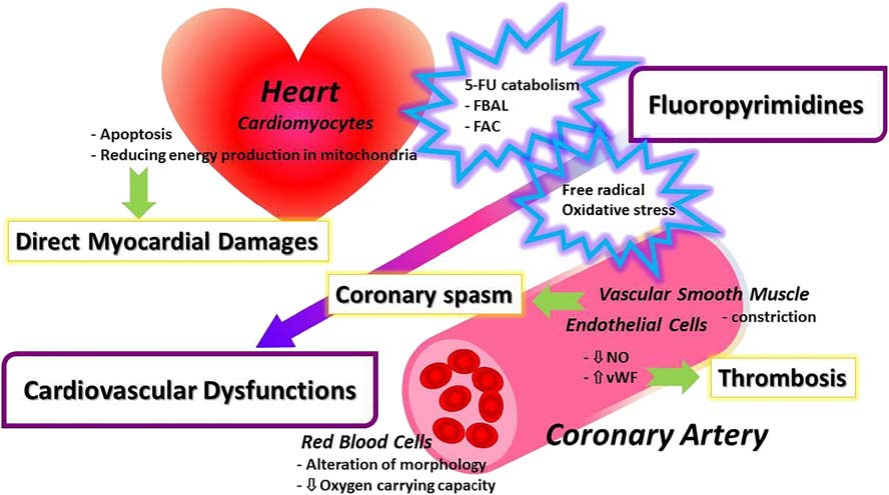
Mechanisms of direct cardiovascular toxicities and coronary spasm by FP. FBAL α-fluoro-β-alanine, FAC fluoroacetate, NO nitric oxide, vWF von Willebrand factor

The pathogenesis and mechanisms of FP-related cardiovascular toxicity development remain to be elucidated [7, 59••]. The reasons for this are multifactorial [60]. The clinical manifestation is coronary spasm in many cases, and vascular endothelial injury is reportedly deeply involved [61••]. Vasoconstriction may also be associated with vascular endothelial injury, involvement of thromboembolism, and myocardial cell damage caused by myocardial ischemia secondary to coronary spasms. In addition, there is direct myocardial cell damage by FP, and the implication of dihydropyrimidine dehydrogenase (DPD) involved in 5-FU catabolism and catabolism products have been reported as the mechanism. Figure 1 shows the outline of the mechanism of FP-related cardiovascular toxicity.

### DPD and its gene (DPYD)

The enzyme first involved in 5-FU catabolism is DPD [62]. Decreases in DPD enzyme activity due to polymorphisms of the gene encoding DPD (*DPYD*) are thought to be associated with common 5-FU-related drug adverse events, including bone marrow suppression and diarrhea. There are reports that *DPYD* mutations are involved in the development of 5-FU related cardiovascular toxicity [63]. On the other hand, there are studies that report no significant associations between *DPYD* mutations and the development of 5-FU related cardiovascular toxicity [64]. One study examined patients with clinical DPD deficiency and found only rare cardiovascular toxicity [62]. Thus, no consensus has been reached regarding this result.

The association between 5-FU dose and cardiovascular toxicity remains unclear [41]. A study examining the relationship between serum 5-FU concentrations and the development of cardiovascular toxicity did not demonstrate a significant relationship [65]. The products of 5-FU catabolism include α-fluoro-β-alanine (FBAL) and fluoroacetate. There are reports that these 5-FU catabolism products may inhibit energy production in the citric acid cycle in mitochondria within myocardial cells, resulting in cardiotoxicity [66, 67]. This is thought to be the mechanism of direct damage to the myocardial cells by FP-related drugs [59••].

### Endothelial dysfunction, vasoconstriction, and thromboembolism

The most important clinical pathophysiology of FP-related cardiovascular toxicity is coronary spasm, in which vascular endothelial cell injury is thought to be markedly involved. There is a report that reversible vasoconstriction involving endothelial dysfunction occurred due to exposure of the aorta to 5-FU in an experimental model [68]. It was reportedly confirmed in experimental analysis that the cellular arrangement of vascular endothelium was collapsed by 5-FU, and thrombi were subsequently formed [69, 70]. Endothelin-1, a peptide derived from vascular endothelial cells, has vasoconstriction properties and is involved in regulation of coronary artery tonus. In patients with 5-FU-related cardiovascular toxicity, plasma endothelin-1 levels were found to be elevated [71]. Protein kinase C is involved in 5-FU-related vasoconstriction, and this enzyme caused endothelial cell-independent vascular smooth muscle constriction in a 5-FU concentration-dependent manner in a rabbit model [72]. Based on these reports, the consensus is that 5-FU causes vascular endothelial cell injury, vasoconstriction involving endothelial cell-dependent mechanisms, induction of coronary spasms, and occasional subsequent thrombus formation, resulting in myocardial dysfunction secondary to myocardial perfusion deficits.

Another study found that 5-FU directly damaged vascular endothelial cells and myocardial cells by blocking cell proliferation cycles [73]. This damage induced oxidative stress and free radical release in endothelial and myocardial cells, leading to apoptosis, culminating in cardiovascular toxicity. Furthermore, 5-FU altered the cell membrane structure of erythrocytes, resulting in reduced oxygen transport capacity and relative ischemia of the myocardium and subsequent cardiac muscle injury [74].

## Treatments

There are no standard recommended drugs based on the evidence for FP-related cardiovascular toxicity. Nevertheless, discontinuation of FP and administration of nitrates and calcium antagonists are thought to significantly improve ischemic symptoms in patients in whom coronary spastic angina clearly developed during FP treatment and in those in whom myocardial ischemia was clearly detected by ECG relative to treatment [75]. When cardiovascular toxicity develops, the suspected drug should be discontinued, and coronary dilators such as nitrates and non-dihydropyridine calcium antagonists should be administered simultaneously. For various cardiovascular toxicities due to FP, including coronary spasm, heart failure, and arrhythmia, it is essential to provide appropriate symptomatic treatment in accordance with recommended treatment guidelines in each country, e.g., the American College of Cardiology/American Heart Association guidelines or the Japanese Circulation Society guidelines [76]. Incidentally, in the Japanese guidelines for the treatment of coronary spastic angina, calcium antagonists are not limited to non-dihydropyridine calcium antagonists. Dihydropyridine calcium antagonists, including nifedipine, benidipine, and amlodipine, are often used in everyday practice of cardiovascular care, and their effects on coronary spasm are well known. Nicorandil has been approved in Japan as a coronary dilator [76].

Uridine triacetate is an oral prodrug of uridine. After uridine is taken up and converted, it reduces the uptake of 5-FU into non-cancer cells, resulting in inhibition of various adverse events, including targeted cell injury and cell death due to 5-FU overdose. Uridine triacetate has been approved by the Food and Drug Administration (FDA) as an antidote for overdose and serious acute adverse events of 5-FU or capecitabine [77]. Nevertheless, there is no sufficient evidence for the effects of uridine triacetate on FP-related cardiovascular toxicity. Future verification is warranted.

## Prophylaxis and preventions, re-challenge

There are mixed opinions regarding whether re-administration of FP to patients who have once developed FP-related cardiovascular toxicity is appropriate. In cases of FP-related cardiovascular toxicity, especially coronary spasm, recurrence of spasm is not uncommon [75]; the reported frequency of cardiovascular toxicity recurrence has been as high as 90% [8, 11, 14, 18, 78–81], and the associated fatality rate was as high as 13% [8]. Therefore, re-challenge is generally not recommended for patients with a history of FP-related cardiovascular toxicity. Even with preventive administration of coronary dilators such as nitrate and calcium antagonists, there is no assurance that prevention of FP-related cardiovascular toxicity is guaranteed [82]. In patients in whom FP re-challenge is absolutely necessary, not only with cooperation with the oncologists but also after thorough examination of the patient’s risk-benefit through multidisciplinary discussion, nitrate and calcium antagonists should be administered prophylactically, and then re-challenge should be considered. Depending on the patient’s disease state and the characteristics of the cancer, the possibility of FP dose reduction and change in drug usage such as switching from continuous intravenous infusion to bolus intravenous injection may be indicated at the time of re-challenge. It is essential to consider treatment on a case-by-case basis. It is not always possible to freely select drugs because of differences in the types of cancers and regions, such as differences by country; nevertheless, it is sometimes possible to consider switching to an alternative FP drug with a low risk of cardiovascular toxicity.

S1 is an oral FP drug consisting of tegafur and gimeracil, which are prodrugs of fluorouracil and oteracil potassium, respectively. Although the use of S1 is not approved in the USA, it is widely used in Japan for cancers including gastric, colon, head and neck, pancreatic, and unresectable and recurrent breast cancers [46]. There are few reports of cardiovascular toxicity by S1. The antagonistic action of gimeracil on DPD may suppress degradation of 5-FU into FBAL, possibly resulting in reduced cardiovascular toxicity [66, 83]. Nevertheless, there is insufficient objective evidence; therefore, the possibility of cardiovascular toxicity by these drugs requires attention in the future.

UFT is an oral combination drug of two agents: tegafur (a prodrug of FU) and uracil (which inhibits FU degradation and increases FU concentrations). The incidence of cardiovascular toxicity is reportedly less than 1% [84, 85]. It can be used in Japan; however, it has not been approved in the USA [46].

TAS-102 is an FDA-approved drug that has been reported to be an FP drug with low cardiotoxicity when administered in patients with colon cancer [86].

## Future directions

As a treatment for FP-related cardiovascular toxicity, experimental evidence suggests that glucagon-like peptide 1 (GLP-1) may counteract 5-FU-induced decreases in the expression levels of endothelial nitric oxide synthase and SIRT-1 indicating its potential as an FP-related cardiovascular toxicity treatment. The possibility of treatment of FP-related cardiovascular toxicity with GLP-1 analogs and GLP-1 degradation inhibitors in the future has also been reported [87•].

Pharmacogenomic interventions are useful to elucidate the relationship between the efficacy of FP drugs and drug-related cardiovascular toxicities and to stratify cardiovascular toxicity risk. Polymorphisms in thymidylate synthase, methylenetetrahydrofolate reductase, and orotate phosphoribosyltransferase, in addition to the *DPYD* gene, are potential risk factors for more serious cardiovascular toxicity [88]. These findings are expected to enable stratification of the cardiovascular toxicity risk prior to FP administration and to select a safe drug administration route, either intravenous infusion or oral. Further investigation in this field is necessary.
